# Supplementary material for: Comparative multiomics analysis of cell physiological state after culture in a basket bioreactor
Source: Sci Rep. 2022 Nov 23;12:20161. doi: 10.1038/s41598-022-24687-4 (PMC9686226; doi:10.1038/s41598-022-24687-4)
Supplement: Supplementary file 1 — Supplementary Information 1. [file 41598_2022_24687_MOESM1_ESM.zip › raw data/Transcriptomic raw data/8.SNP/3.stat/SNP_function.pdf]

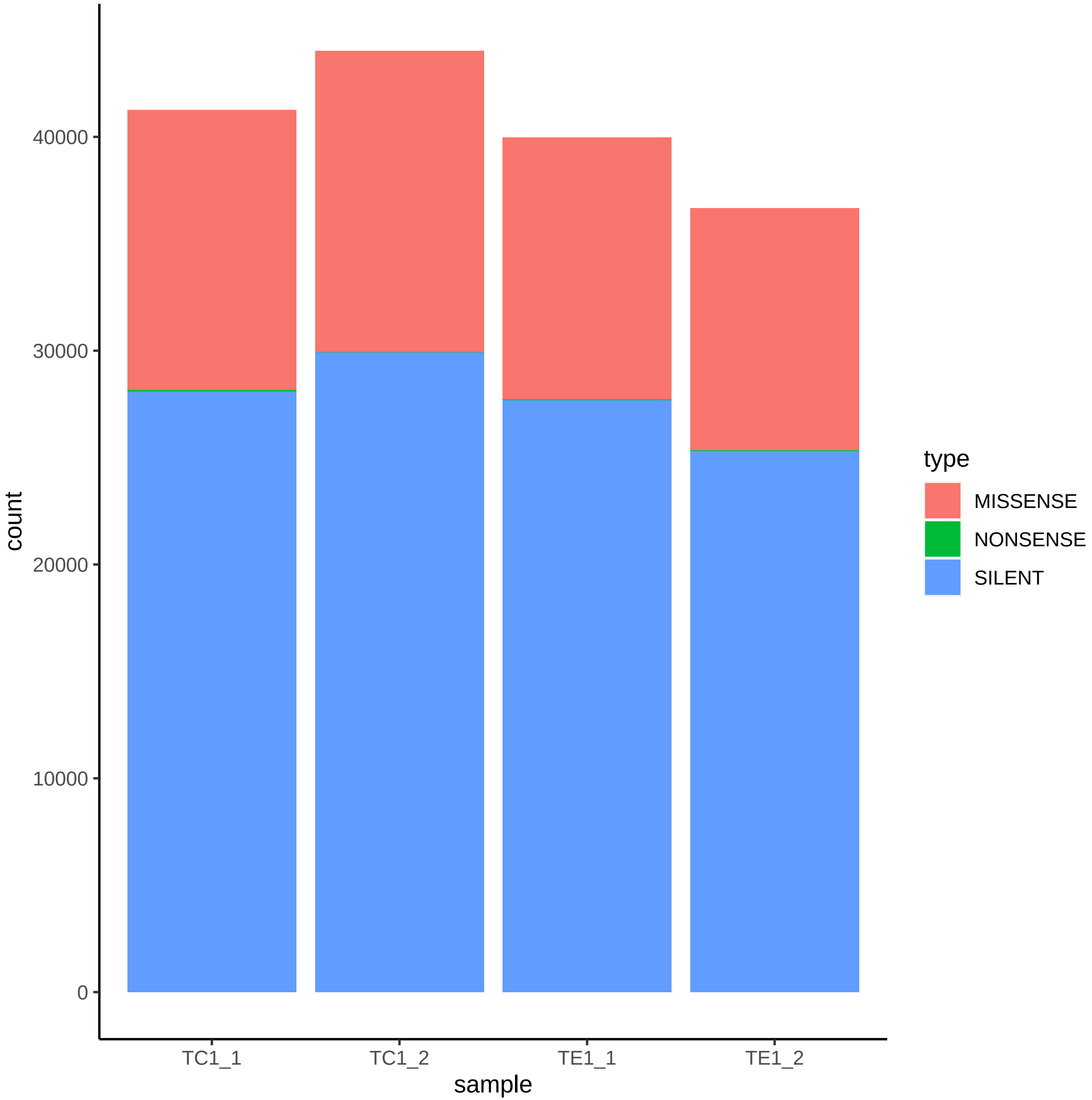
count
40000
30000
20000
10000
0
TC1_1
TC1_2
TE1_1
TE1_2
sample
type
MISSENSE
NONSENSE
SILENT
